# Herpesvirus Regulation of Selective Autophagy

**DOI:** 10.3390/v13050820

**Published:** 2021-05-01

**Authors:** Mai Tram Vo, Young Bong Choi

**Affiliations:** Department of Oncology, Sidney Kimmel Comprehensive Cancer Center, Johns Hopkins University School of Medicine, Baltimore, MD 21287, USA; mvo8@jhmi.edu

**Keywords:** herpesviruses, autophagy, selective autophagy, mitophagy, aggrephagy, ferritinophagy, nucleophagy, virophagy

## Abstract

Selective autophagy has emerged as a key mechanism of quality and quantity control responsible for the autophagic degradation of specific subcellular organelles and materials. In addition, a specific type of selective autophagy (xenophagy) is also activated as a line of defense against invading intracellular pathogens, such as viruses. However, viruses have evolved strategies to counteract the host’s antiviral defense and even to activate some proviral types of selective autophagy, such as mitophagy, for their successful infection and replication. This review discusses the current knowledge on the regulation of selective autophagy by human herpesviruses.

## 1. Introduction

Macroautophagy (simply called autophagy) is a conserved cellular degradation and recycling system that is essential for cell survival during normal physiological conditions and in response to different types of stress, such as starvation [1]. In its common form, autophagy proceeds through the formation of double-membrane vesicles, termed autophagosomes, that engulf portions of the cytoplasm and deliver them to lysosomes. In addition to the non-selective autophagy, forms of selective autophagy have evolved to remove specifically damaged, superfluous, or unwanted materials including protein aggregates and subcellular organelles, thereby providing cellular quality control and homeostasis [2,3,4]. Selective autophagy is mediated by autophagy cargo receptors, which contain an LC3-interacting region (LIR) [5] and can therefore bind directly to the autophagy-related gene 8 (ATG8) family proteins, including MAP1LC3A (LC3A), LC3B, LC3C, GABARAP, GABARAPL1, and GABARAPL2, on the phagophore [6].

Various selective autophagy pathways have been characterized and classified according to the type of targeted cellular materials (Table 1) [7]: for example, protein aggregates (aggrephagy), mitochondria (mitophagy), endoplasmic reticulum (ER-phagy or reticulophagy), nuclei (nucleophagy), ribosomes (ribophagy), lipid droplets (lipophagy), lysosomes (lysophagy), peroxisomes (pexophagy), ferritin (ferritinophagy), nucleic acids in the cytosol (DN-autophagy and RN-autophagy), and intracellular pathogens (xenophagy). In addition, based on the type of autophagy receptor and cargo-targeting mechanisms, selective autophagy is also subdivided into ubiquitin-dependent and independent pathways. Ubiquitin-dependent selective autophagy is mediated by sequestosome-like receptors (SLRs) (Table 1), including p62/SQSTM1, NBR1, optineurin (OPTN), NDP52/CALCOCO2, and TAX1BP1 that contain a ubiquitin-binding motif, that recognize intracellular ubiquitinated substrates on mitochondria, bacteria, peroxisomes, and aggregated proteins [7]. On the other hand, Ub-independent selective autophagy relies on autophagy receptors targeted directly to substrates that, under certain conditions, bridge their cargo to ATG8-containing autophagosome membranes. These receptors include BNIP3, NIX/BNIP3L, FUNDC1, TBC1d5, PHB2, STBD1, and FAM134B (Table 1) [7].

Selective autophagy is known to regulate and be regulated by virus infection. In general, it is believed that the host cells use autophagy as a defense against invading viruses. Viral xenophagy, simply termed virophagy, recognizes whole virion particles or virion components for clearance (Table 1), but it is largely unknown how viruses are recognized by selective autophagy receptors or associated factors. On the other hand, viruses have evolved elaborate mechanisms to evade antiviral autophagy, or even to activate certain forms of selective autophagy for successful infection or replication. The latter case is exemplified by mitophagy, which attenuates mitochondria-mediated antiviral responses, including apoptosis and innate immune responses. Several articles have reviewed in detail the mechanisms by which viruses evade antiviral autophagy, in particular, acting on the autophagy core machinery [8,9,10,11,12,13,14,15]. Here, we describe the regulation of selective autophagy, including mitophagy, aggrephagy, nucleophagy, ferritinophagy, and virophagy, by herpesviruses.

## 2. Mitophagy

Mitochondria have been known to act as a platform for antiviral signaling that leads to apoptosis and type-I interferon (IFN) expression in response to virus infection [16,17]. Therefore, a variety of RNA and DNA viruses have evolved strategies to attenuate mitochondria-mediated antiviral responses for their own benefit [13,16,17,18]. On the other hand, mitochondria normally generate reactive oxygen species (ROS) from the mitochondrial respiratory chain reaction [19]. Accumulation of damaged or dysfunctional mitochondria leads to the generation of excessive ROS, which can potentiate apoptosis through mitochondrial membrane depolarization and augment innate immune signaling [20,21,22]. Thus, viruses have to eliminate altered mitochondria to promote their successful infection and replication. Indeed, recent studies have demonstrated that RNA and DNA viruses can eliminate infection-altered mitochondria via mitochondria quality control, which is achieved by balanced actions among mitochondrial biogenesis, dynamics (fission and fusion events), and mitophagy, potentially related to attenuation of apoptosis and the innate immune response (Table 2).

Mitophagy is a selective form of autophagy that eliminates dysfunctional or superfluous mitochondria in a ubiquitin-dependent or independent manner [42]. The mitochondria-localized kinase PINK1 and the ubiquitin (Ub) E3 ligase Parkin (PARK2) are the best known mitophagy-associated proteins. Upon the loss of mitochondrial membrane potential, the PINK1 and Parkin complex induces ubiquitination of mitochondria [43], which is recognized by ubiquitin-binding SLR autophagy receptors [44] and then targeted to autophagosomes via interaction with ATG8 family proteins. In addition, the ubiquitination-independent mitophagy pathways via mitophagy receptors, such as NIX/BNIP3L, BNIP3, FUNDC1, and PHB2, have been identified under certain physiological and pathological conditions, including erythrocyte differentiation, paternal mitochondrial elimination, hypoxia, and virus lytic replication [41,45,46,47,48]. It is believed that mitochondrial fission/fragmentation is prerequisite for mitophagic removal of damaged parts of mitochondria. Here, we describe recent findings on the regulation of mitochondrial fragmentation and mitophagy by human herpesviruses. Most herpesviruses, except for human herpesvirus 8 (see below), have not been proven to activate mitophagy receptors directly from their gene products, but we discuss some human herpesviruses showing activity which induces mitochondrial fragmentation, demonstrating their potential for mitophagy activation.

### 2.1. Herpes Simplex Virus 1 (HSV-1) and Herpes Simplex Virus 2 (HSV-2)

HSV-1, also known as human herpesvirus 1 (HHV-1), and HSV-2, also known as HHV-2, are so ubiquitous that two third of the human population in the world are infected by at least one of these viruses [49]. Although infections by HSV-1 and HSV-2 are usually asymptomatic or associated with mild symptoms, the viruses have been considered as causative agents in severe neuronal diseases, such as herpes simplex encephalitis (HSE) and meningitis [50]. The severe manifestations are postulated to be associated with mitochondrial dysfunction in cultured neuron cells after HSV-1 and HSV-2 infection [51]. HSV-1 and HSV-2 infection-induced mitochondrial dysfunction occurs at multiple levels, including upregulation of mitochondrial fission, decrease in the mitochondrial membrane potential, and increase in ROS levels. The changes in the organization of the mitochondrial network in infected neurons provide appropriate conditions for HSV-1 and HSV-2 replication and are required for effective viral dissemination [51]. Furthermore, in brain tissues of HSE cases, the levels of mitochondrial DNA (mtDNA) and mtDNA-encoded proteins are significantly reduced, suggesting that mitophagy may be activated following HSV-1 infection in neurons. Together, these results suggest that HSV-1 and HSV-2 may activate mitochondrial quality control mechanisms for their productive replication, and that this may contribute to pathogenesis. However, there is no direct evidence to date that HSV-1 and HSV-2 activate mitophagy.

### 2.2. Varicella-Zoster Virus (VZV)

Primary infection by VZV, also known as HHV-3, causes varicella (chickenpox) during which VZV becomes latent in ganglionic neurons. When reactivated, it causes zoster (shingles), which can be complicated by chronic pain and other serious neurological disorders, such as meningoencephalitis, myelitis, and vasculopathy [52]. Although one report showed that VZV infection induces a time-dependent significant change in mitochondrial morphology (fragmentation), potentially via its immediate-early gene product IE63, in human fetal lung cells [53], whether mitophagy activation is associated with VZV infection and associated pathogenesis has not been demonstrated.

### 2.3. Epstein-Barr Virus (EBV)

Infection by EBV, also known as HHV-4, causes various human malignancies, including posttransplant lymphoproliferative diseases, nasopharyngeal carcinoma, Hodgkin’s lymphoma, Burkitt lymphoma, and gastric carcinoma [54]. The EBV-encoded product BHRF1, a BCL2 homolog, is known to activate mitophagy via modulating mitochondrial dynamics or mitochondrial membrane permeabilization transition (MMPT) in BHRF1-transfected cervical (HeLa) or nasopharyngeal (CNE1 and 5-8F) cancer cell lines and EBV-reactivated AKATA cells [55,56]. BHRF1 expression stimulates DRP1-mediated mitochondrial fission and, concomitantly, autophagic flux by interacting with Beclin 1, thereby attenuating type-I IFN induction [55]. The BHRF1-induced mitochondrial fission and autophagy stimulation lead to mitochondrial network reorganization to form juxtanuclear mitochondrial aggregates, which then cause the induction of mitophagy and the accumulation of PINK1 at the mitochondria. In addition, BHRF1 expression is known to induce cyclophilin D-dependent MMPT, which in turn increases ROS production and activates mitophagy in nasopharyngeal carcinoma cell lines [56]. In addition, the viral latent membrane protein 2A (LMP2A) has been shown to promote DRP1-mediated mitochondrial fission in stably transfected gastric and breast cancer cell lines, AGS and MCF7 respectively, suggesting that LMP2A-induced mitochondrial fission may contribute to tumorigenesis of EBV-associated epithelial cancers [57]. Overall, BHRF-1- or LMP2A-regulated mitochondrial dynamics and mitophagy are implicated in the promotion of EBV tumorigenesis [56].

### 2.4. Human Cytomegalovirus (HCMV)

HCMV, also known as HHV-5, infects most individuals in the world, mostly without showing overt symptoms. Modern advanced diagnostic methods reveal that HCMV is a common opportunistic infection in fetuses and immunocompromised individuals, including organ transplant patients and AIDS patients [58]. It is not known if mitophagy is activated in response to HCMV infection. Instead, HCMV infection promotes mitochondrial biogenesis and respiration to support bioenergetic and biosynthetic requirements early, during the viral replication cycle in human foreskin fibroblasts and U373 astrocytoma cells [59,60]. The immediate-early protein pUL37x1, also known as the viral mitochondrial inhibitor of apoptosis (vMIA), is known to induce mitochondrial fragmentation in human fibroblasts [61,62,63]. However, it is unclear to date whether pUL37x1-induced mitochondrial dynamics are associated directly with the inhibition of BAX-dependent or -independent apoptosis [64,65], or with HCMV infection-induced mitochondrial biogenesis. Future studies are warranted to understand how HCMV infection or pUL37x1 regulates mitochondrial quality control in infected cells.

### 2.5. Human Herpesvirus 8 (HHV-8)

HHV-8, also known as Kaposi’s sarcoma-associated herpesvirus (KSHV), is the etiological agent that is causally associated with at least three human malignancies, Kaposi’s sarcoma (KS), primary effusion lymphoma (PEL), and multicentric Castleman’s disease (MCD) in immunodeficient individuals with HIV infection or AIDS [66,67]. Like other herpesviruses, HHV-8 has two distinct infection stages in the host: latency (persistent infection) and lytic replication, both of which are implicated in HHV-8 pathogenesis. In particular, PEL and MCD are likely associated with lytic replication [68,69,70]. Recently, it has been demonstrated that HHV-8-encoded viral IFN regulatory factor 1 (vIRF-1) localizes in part to mitochondria, where it interacts directly with the mitophagy receptor NIX and promotes NIX-mediated mitophagy to remove dysfunctional mitochondria during lytic replication in HHV-8-infected PEL cells [41]. vIRF-1 also has the ability to induce DRP1-dependent mitochondrial fragmentation in transfected HeLa cells [41], which may contribute to the promotion of mitophagy. Although, the exact mechanisms by which vIRF-1 orchestrates mitochondrial dynamics to induce mitophagy remains to be determined. This pro-mitophagic activity of vIRF-1 is linked to the inhibition of apoptosis and the promotion of virus productive replication.

## 3. Aggrephagy

It is believed that soluble misfolded proteins are repaired or removed by protein quality control machineries, such as folding complex-associated chaperons and the ubiquitin-proteasome-mediated degradation system (UPS), respectively. Unfolded or misfolded proteins in the lumen of the endoplasmic reticulum (ER) are resolved by the unfolded protein response (UPR) that enhances the expression of ER chaperones and components of ER-associated degradation [71]. When misfolded proteins fail to be rescued, however, they form protein aggregates, which are then removed by a selective form of autophagy, called aggrephagy [72]. Aggrephagy is mediated by autophagy receptors, TOLLIP, a human homologue of *Saccharomyces* Cue5 that contains a CUE domain, and specific SLRs, including p62/SQSTM1, NBR1, and OPTN [73,74,75,76,77]. Intriguingly, OPTN-mediated aggrephagy is independent of ubiquitin [77]. In addition, WDFY3, also known as ALFY [78], is involved in the clearance of aggregated polyQ proteins by interacting with a number of different partners, including p62/SQSTM1 [79], GABARAP subfamily members [80], and phosphatidylinositol-3-phosphate [81], a prominent lipid in the regulation of autophagosome membrane formation. These results suggest that WDFY3 acts as a scaffold protein in p62/SQSTM1-dependent degradation of ubiquitinated aggregates. Interestingly, in yeast, the mechanistic determination of protein removal by aggrephagy versus degradation appears to be independent of the ubiquitin-binding properties of the receptors and largely determined by their oligomerization potentials [82]. In fact, oligomerization of p62/SQSTM1 and Cue5 is required for selective autophagy of ubiquitinated cargo [83,84]. Therefore, it is likely that WDFY3 promotes the oligomerization of p62/SQSTM1 for aggrephagy activation.

Recently, aggrephagy was proposed to play a proviral role in herpesvirus infection. Murine cytomegalovirus (MCMV)-encoded M45 protein, a potent cell death inhibitor [85], interacts with two cellular signaling proteins, NEMO and RIPK1, and induces their aggregation via the induced protein aggregation motif (IPAM) of M45 [86]. The aggregation of NEMO blocks NF-ĸB signaling, and the aggregation of RIPK1 blocks necroptosis, thereby contributing to immune evasion and cell viability, and to increased virus replication in mouse embryonic fibroblasts (MEF) [86]. Mechanistically, the complex of M45 with NEMO and RIPK1 recruits the retromer component vacuolar protein sorting 26B (VPS26B) and TBC1 domain family member 5 (TBC1d5), the latter containing two LC3-interacting region (LIR) motifs, to facilitate the selective autophagy.

Several human herpesviruses encode M45 homologues containing the IPAM motif: HSV-1 ICP6, HSV-2 ICP10, EBV BORF2, and HHV-8 ORF61 [86]. Among them, HSV-1 ICP6, a viral ribonucleotide reductase [87], was demonstrated experimentally to have comparable activity to M45 in aggrephagy of RIPK1 in human foreskin fibroblasts [86]. Future studies are required to determine whether the other M45 homologues have similar aggrephagy-inducing activities during virus infection or replication.

## 4. Nucleophagy

Nucleophagy is a selected form of autophagy that removes nuclear components [88]. While autophagic degradation of entire nuclei was reported in the filamentous fungus *Aspergillus oryzae* that has multi-nucleated hyphae [89], it is unlikely to occur in any mononuclear cell, as this would be lethal. Some studies have suggested that selective autophagic degradation of chromatin and nuclear lamina could play a role in preventing tumorigenesis [90,91].

Autophagic degradation of nuclear materials may be either proviral or antiviral in herpesvirus infection. The nucleus is the site of herpesvirus latency and replication. Therefore, it is conceivable that the host cell may activate nucleophagy to directly remove herpesvirus genomic DNA or proteins, located within the nucleus, that are essential for infection. However, such antiviral autophagy has not been reported to date. Instead, autophagic epitope processing of EBV EBNA1, a nuclear protein that maintains the viral genome of EBV as an episome in the host cell nucleus, was reported to be involved in MHC class II presentation of EBNA1 for acquired immune response to EBV-infected cells [92]. In fact, EBNA1 is the first viral protein identified as being degraded by autophagy. However, whether EBNA1 is recognized by the selective autophagy machinery, such as p62 or LC3B, in the nucleus or the cytoplasm after nuclear export, is unknown. Thus, the precise mechanism by which EBNA1 is processed by autophagy remains to be elucidated.

On the other hand, EBV BFRF1, a major component of nuclear egress complexes, is expressed at the early stage of lytic replication of EBV, localizes at the nuclear envelope, and facilitates the translocation of vesicles from the nucleus to cytoplasm [93,94,95,96]. Thus, some aggregated nuclear proteins, which may be harmful to virus-infected cells, can be engulfed by the BFRF1-derived vesicles and moved to the cytoplasm, where they are degraded in an autophagy-dependent manner [97], suggesting that BFRF1-mediated nucleophagy plays a proviral role in EBV productive replication. Mechanistically, WDFY3 and p62/SQSTM1, which are involved in aggrephagy activation as mentioned above, recognize aggregated nuclear proteins and facilitate BFRF1-induced protein degradation. It is noteworthy that BFRF1 is found to have an important role for virion egress from the nucleus via BFRF1-derived vesicles [98]. Therefore, it is necessary to determine how BFRF1-mediated trafficking of EBV virions evade autophagic degradation. On the other hand, it is known that autophagic degradation of nuclear laminar proteins, including lamins A/C, B1, and B2, facilitates the nuclear egress of HSV-1 in permissive immature dendric cells (DCs), but not mature DCs [99], suggesting that nucleophagy is required for HSV-1 replication in a cell-type dependent manner.

## 5. Ferritinophagy

Ferritinophagy is a selected form of autophagy that degrades the iron-sequestering protein ferritin [100]. Iron is a crucial component of various enzymes and proteins, making it essential for several cellular processes. However, excessive free iron induces oxidative stress by promoting ROS production and is harmful to the cell [101]. Ferritin acts as a buffer for iron when cellular iron levels are high. Conversely, when iron levels are low, ferritin is degraded by ferritinophagy to release iron [100]. Upon iron depletion, nuclear receptor coactivator 4 (NCOA4) is stabilized, specifically binds to the heavy chain of ferritin, and marks ferritin as autophagic cargo, allowing it to be selectively degraded [102]. However, NCOA4 has no conventional LIR motif, which is found in other autophagy receptors and required for binding to the ATG8-containing phagophore membrane. Interestingly, a recent study discovered that the NCOA4-ferritin complex is targeted to lysosomes via the non-canonical autophagy pathway mediated by ATG9A and VPS34 proteins [103].

Ferritinophagy is proposed as a potential host antiviral response to HCMV infection. A recent study showed that premature cell death induced by HCMV infection in MRC5 human lung fibroblasts is inhibited by its gene product pUL38, which interacts with the host protein ubiquitin-specific protease 24 (USP24) and then leads to a decrease in NCOA4 stability, thereby resulting in the inhibition of ferritinophagy [104]. Consistent with this, blockage of ferritinophagy by genetic ablation of NCOA4 prevented pUL38-deficient HCMV infection-induced cell death [104]. Therefore, pUL38 plays an important role in the inhibition of NCOA4-mediated ferritinophagy, leading to increased cell viability and successful virus infection.

## 6. Viral Xenophagy (Virophagy)

Xenophagy is a common term used for selective autophagic degradation of intracellular pathogens, including bacteria, viruses, and fungi, which comprises an important part of the host immune response [105]. Therefore, viral xenophagy (virophagy) refers to the host antiviral response that removes virions or, conceivably, viral components (proteins or genetic materials) in response to virus infection or replication. Following bacterial infection, proteins on the surface of bacteria are rapidly ubiquitinated and recognized by SLRs, including p62/SQSTM1, NDP52, and OPTN [106,107,108]. However, the molecular mechanisms by which whole virion particles are recognized and targeted to autophagosomes by the autophagy machinery have been relatively understudied.

A recent genome-wide screening study showed that the DNA repair genes, Fanconi anemia complementation group A (FANCA) and C (FANCC), are involved in the autophagic degradation of Sindbis virus (enveloped RNA virus) as well as HSV-1 [109]. Importantly, Sumpter and colleagues demonstrated that FANCC binds biochemically to the Sindbis virus capsid protein, indicating that FANCC recognizes the de-enveloped nucleocapsid of Sindbis virus in the cytosol [109]. However, it is not known whether FANCC binds to the nucleocapsid or tegument proteins of HSV-1, whereas an in vivo study verified that FANCC-deficient mice are more susceptible to lethal central nervous system infection by HSV-1, compared to wild-type mice [109]. It is noteworthy that the study used the HSV-1 mutant virus (ICP34.5^Δ68–87^), in which amino acids 68–87 required for Beclin 1 binding of and autophagy inhibition by the HSV-1 neurovirulence protein ICP34.5 is deleted. On the other hand, FANCC is recruited to mitochondria and interacts with Parkin for mitophagy activation, indicating that FANCC-activated mitophagy is dependent on ubiquitination [109]. However, it is not known if FANCC-mediated virophagy is dependent on ubiquitination. An image-based genome-wide siRNA screen found that the HECT-type E3 ubiquitin ligase SMURF1 is required for the autophagic targeting of both Sindbis and HSV-1 viruses [110]. Therefore, it is likely that FANCC and SMURF1 physically and/or functionally interact each other to facilitate virophagy. Intriguingly, a recent genome-wide short interfering RNA screen study showed that virion-containing endosomes can be targeted for virophagy. The endosomal protein sorting nexin 5 (SNX5) interacts with ATG14-containing class III phosphatidylinositol-3-kinase complex 1 (PI3KC3-C1) upon HSV-1 (ICP34.5^Δ68–87^) infection and increases the lipid kinase activity of PI3KC3-C1, which in turn promotes endosomal generation of phosphatidylinositol-3-phosphate (PtdIns(3)P), and recruitment of (PtdIns(3)P)-binding protein WIPI2 to virion-containing endosomes [111].

In addition to the autophagic recognition of virions, viral components, such as proteins and the genomes of RNA and DNA viruses, may be recognized by autophagy receptors or associated proteins for degradation. DN-autophagy and RN-autophagy are selected forms of autophagy that remove DNA and RNA, respectively, in the cytosol [112]. The lysosomal proteins LAMP2C and SIDT2 are known to bind to both cytosolic DNA and RNA and mediate the direct transport of nucleic acids into lysosomes [113,114]. However, to date, it is unknown whether viral RNA and DNA in the cytosol are cleared by selective autophagy. Interestingly, HSV-1 infection-induced autophagy in macrophages is known to be dependent on STING (stimulator of IFN genes) [115], which is an adaptor protein that is activated by cyclic guanosine monophosphate–adenosine monophosphate (cGAMP), produced by the DNA sensor cGAMP synthase [116]. STING-mediated autophagy is known to restrict HSV-1 infection independently of a STING-mediated innate immune response that leads to the expression of type-I IFNs [117]. In fact, RNA interference-mediated knockdown of TBK1 and IRF3, key components of innate immunity, does not affect STING-mediated autophagy in HeLa cells [118]. Instead, STING dimerization and binding to LC3 via its LIR motifs are required for autophagy activation [118]. Overall, these results suggest that viral DNA in the cytosol can elicit the host antiviral autophagy mediated by STING.

On the other hand, the role of TBK1 in STING-mediated autophagy seems controversial in HSV-1 infection [118,119]. A recent study demonstrated that TBK1 is required for early autophagy induction, in a STING-dependent manner, upon HSV-1 infection using TBK1^+/−^ dermal fibroblasts from herpes simplex encephalitis with autosomal dominant TBK1 containing a dominant-negative G159A mutation [119]. Interestingly, Ahmad and colleagues found two LC3B phenotypes: perinuclear LC3B puncta (also known as nuclear envelope-derived autophagy, NEDA) and cytoplasmic LC3B puncta. The latter puncta are formed early upon HSV-1 infection or cyclic diguanylate monophosphate (c-di-GMP) transfection in wild-type, but not TBK1^+/−^ dermal fibroblasts and involved in cytoprotection from HSV-1 infection. This study highlights a possibly cytoprotective role for TBK1 in HSV-1-induced cytoplasmic autophagy. Other evidence supports the role of TBK1 in autophagy-mediated restriction of HSV-1; tripartite motif protein 23 (TRIM23), a RING-type E3 ubiquitin ligase with ADP-ribosylation factor (ARF) GTPase activity, is known to activate TBK1 on the autophagosome membranes, which in turn phosphorylates p62 to induce autophagy [120,121]. Overall, TBK1 requirement of STING-mediated autophagy may be dependent on cell type.

## 7. Viral Evasion of Antiviral Autophagy

Viruses have evolved the strategies to evade antiviral autophagy or virophagy by targeting the autophagy core machinery [8,9,10,11]. For example, the HSV-1 neurovirulence protein ICP34.5 prevents formation of the phagophore by binding to Beclin 1, a key factor involved in the elongation of the phagophore membrane, and by redirecting the protein phosphatase 1α (PP1α) to dephosphorylate translation initiation factor 2α (eIF2α) in neuron cells and fibroblasts [122,123]. The Us11 protein is also known to inhibit autophagy by direct interacting with and inhibiting the double-stranded RNA-dependent protein kinase PKR in HeLa cells and fibroblasts, thereby reducing the level of phosphorylated eIF2α [124]. In addition, the ICP0 protein downregulates SLR autophagy receptors p62/SQSTM1 and OPTN during the early stages of HSV-1 infection in various cell lines, including human embryonic lung fibroblasts (HEL), human epithelial cells (HEp-2), human microglia cell line 3 (HMC3), human osteosarcoma cell line (U2OS), but not human monocyte cell line (THP1) [125]. Furthermore, the Us11 protein excludes TBK1 from the TRIM23/HSP90 complex by binding to the ARF domain of TRIM23, thereby inhibiting autophagy-mediated restriction of HSV-1 infection in MEF and HEL [126]. These results indicate that HSV-1 has developed multiple strategies to incapacitate autophagy to promote virus replication. For HCMV, autophagy is rapidly induced upon virus infection in MRC5 cells but inhibited by the HCMV-encoded proteins TRS1 and IRS1, which blocks autophagosome biogenesis by interacting with Beclin 1 [127]. For HHV-8, the viral proteins vFLIP and vBcl-2 can suppress autophagy by preventing ATG3 from binding and processing LC3 [128], and by inhibiting Beclin 1 [129], respectively.

## 8. Conclusions

**Figure 1:**
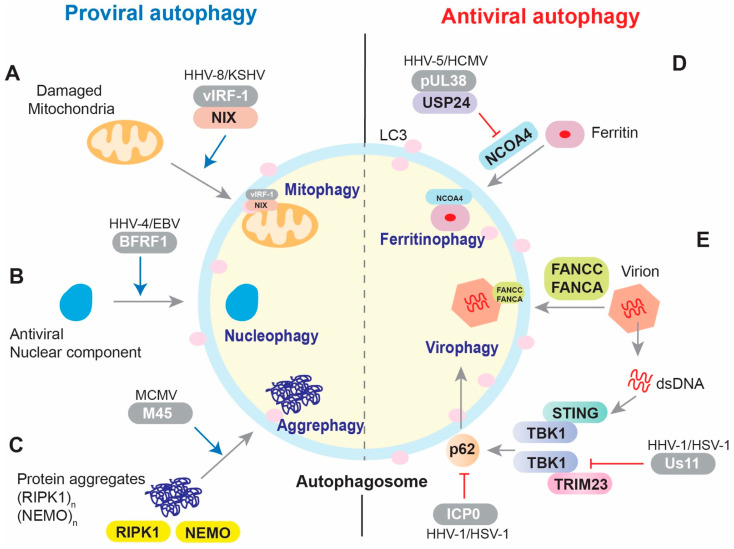
Overview of the regulation of selective autophagy by herpesviruses. (**A**) Mitophagy. HHV-8 vIRF-1 activates NIX-mediated mitophagy during lytic replication. (**B**) Nucleophagy. EBV BFRF1 promotes nuclear vesicle-mediated autophagy following reactivation. (**C**) Aggrephagy. MCMV M45 promotes the aggregation of RIPK1 and NEMO and then autophagic clearance of the aggregated proteins. (**D**) Ferritinophagy. HCMV pUL38 inhibits NCOA4-mediated autophagy by interacting with USP24, which destabilizes NCOA4. (**E**) Virophagy. The Fanconi anemia proteins FANCA and FANCC mediate autophagic clearance of HSV-1. In addition, p62/SQSTM1-mediated virophagy can be activated by TRIM23 or STING via TBK1 after HSV-1 infection. The ICP0 protein downregulates the autophagy receptor p62/SQSTM1 during the early stages of HSV-1 infection, and the Us11 protein excludes TBK1 from the TRIM23/HSP90 complex to inhibit autophagy-mediated restriction of HSV-1 infection. Viral proteins are highlighted within gray capsules.

A variety of selective autophagy mechanisms have been identified, and more will be revealed upon the discovery of specific cargo and autophagy receptor partners. Selective autophagy contributes to cellular homeostasis via quality and quantity control of the specific cargo. Viruses have evolved strategies to activate or inhibit selective autophagy for their own benefit, that is, to achieve successful infection and replication (Figure 1). Complex and varied mechanisms of herpesvirus regulation of autophagy are likely a consequence of cell-type specificities, infection type (*de novo*, lytic, latent), kinetic stage, and particular subfamily associated herpesvirus biologies. In this paper, we have focused on the molecular mechanisms and functional implications of herpesvirus regulation of mitophagy, aggrephagy, nucleophagy, ferritinophagy, and virophagy. In addition to elucidating further details about these processes, it is important to also investigate the role and regulation of other selective autophagy pathways, including pexophagy, lysophagy, lipophagy, reticulophagy, and glycophagy, in herpesvirus infection and replication.

## Figures and Tables

**Table 1:** Selective autophagy and viruses.

Selective Autophagy (SA)	Targeted Cargos	SA Receptors and Associated Factors	Effects on Herpesvirus Infection
Mitophagy	Mitochondria	SLRs ^1^ (p62, NDP52, OPTN, NBR1, and TAX1BP1)	Proviral
		BNIP3, NIX/BNIP3L, FUNDC1, PHB2	
		
Aggrephagy	Aggregated proteins	SLRs (p62 and NBR1)	Proviral
		TOLLIP, TBC1d5	
Nucleophagy	Nuclei	SLRs (p62)	Proviral
Ferritinophagy	Ferritin	NCOA4	Antiviral
Xenophagy/Virophagy (viruses)	Intracellular viruses	SLRs (p62) STING, TRIM23, TBK1, FANCA/FANCC	Antiviral
Xenophagy (bacteria)	Intracellular bacteria	SLRs (p62, NDP52, and OPTN)	
Reticulophagy	Endoplasmic reticulum	RETREG1/FAM134B	
Pexophagy	Peroxisomes	SLRs (p62 and NBR1)	
Ribophagy	Ribosomes	NUFIP1	
Lipophagy	Lipids (Lipid drops)	AUP1	
Lysophagy	Lysosomes	SLRs (p62)	
Glycophagy	Glycogen	STBD1	
DN- and RN-autophagy	Nucleic acids	LAMP2C, SIDT2	

^1^ SLRs: Sequestosome-like receptors.

**Table 2:** Viral regulation of mitochondrial dynamics and mitophagy.

Genome	Viruses Name	Viral Proteins	Mechanisms of Action	Effects	Refs
RNA	Influenza A virus (IAV)	PB1-F2	TUFM-mediated mitophagy	Evasion of innate immune response	[23]
	Human parainfluenza virus (HPIV3)	M protein	TUFM-mediated mitophagy	Evasion of innate immune response	[24]
	Hantavirus	Gn protein	TUFM-mediated mitophagy	Evasion of innate immune response	[25]
	Hepatitis C virus (HCV)	Not known	DRP1-mediated mitochondrial fission/ Parkin-mediated mitophagy	Promotion of virus replication	[26,27]
		NS5A	PINK1/parkin-mediated mitophagy	Promotion of cell survival	[28]
	Humanimmunodeficiency virus (HIV)	gp120 and Tat	DRP1-mediated mitochondrial fission/ Parkin-mediated mitophagy	Promotion of virus replication	[29]
	Coxsackievirus B (CVB)	Not known	DRP1-mediated mitochondrial fission	Promotion of virus productiveinfection	[30]
	Classical swine fever virus (CSFV)	Not known	Mitochondrial fission/ PINK1/parkin-mediated mitophagy	Inhibition of apoptosis	[31]
	Transmissible gastroenteritis virus(TGEV)	Not known	Inducing mitophagy	Inhibition of apoptosis	[32]
	Porcine reproductive and respiratory syndrome virus (PRRSV)	Not known	DRP1-mediated mitochondrial fission/ Parkin-mediated mitophagy	Inhibition of apoptosis	[33]
	Newcastle disease virus (NDV)	Not known	p62-mediated mitophagy	Inhibition of apoptosis	[34]
DNA	BK-polyomavirus (BKV)	Agnoprotein	p62-mediated mitophagy	Evasion of innate immune response	[35]
	Porcine circovirus 2 (PCV2)	Capsid (?)	ROS production/ DRP1 phosphorylation/ PINK1/parkin-mediated mitophagy	Promotion of apoptosis (?)	[36]
	Bovine papillomaviruses (BPVs)	E5	PINK1/parkin-, BNIP3-, NIX-, and FUNDC1-mediatedmitophagy	Promotion of virus replication	[37,38,39]
	Hepatitis B virus (HBV)	HBx	DRP1 phosphorylation/ PINK1/parkin-mediated mitophagy	Inhibition of apoptosis	[40]
	Human herpesvirus 8 (HHV-8)	vIRF-1	NIX-mediated mitophagy	Inhibition of apoptosis/ Promotion of virus productive infection	[41]

## References

[1] Mizushima N. (2007). Autophagy: Process and function. Genes Dev..

[2] Okamoto K. (2014). Organellophagy: Eliminating cellular building blocks via selective autophagy. J. Cell Biol..

[3] Knaevelsrud H., Simonsen A. (2010). Fighting disease by selective autophagy of aggregate-prone proteins. FEBS Lett..

[4] Galluzzi L., Baehrecke E.H., Ballabio A., Boya P., Bravo-San Pedro J.M., Cecconi F., Choi A.M., Chu C.T., Codogno P., Colombo M.I. (2017). Molecular definitions of autophagy and related processes. EMBO J..

[5] Wild P., McEwan D.G., Dikic I. (2014). The LC3 interactome at a glance. J. Cell Sci..

[6] Schaaf M.B., Keulers T.G., Vooijs M.A., Rouschop K.M. (2016). LC3/GABARAP family proteins: Autophagy-(un)related functions. FASEB J..

[7] Khaminets A., Behl C., Dikic I. (2016). Ubiquitin-Dependent And Independent Signals In Selective Autophagy. Trends Cell Biol..

[8] Yin H.C., Shao S.L., Jiang X.J., Xie P.Y., Sun W.S., Yu T.F. (2019). Interactions between Autophagy and DNA Viruses. Viruses.

[9] Vescovo T., Pagni B., Piacentini M., Fimia G.M., Antonioli M. (2020). Regulation of Autophagy in Cells Infected With Oncogenic Human Viruses and Its Impact on Cancer Development. Front. Cell Dev. Biol..

[10] Lussignol M., Esclatine A. (2017). Herpesvirus and Autophagy: “All Right, Everybody Be Cool, This Is a Robbery!”. Viruses.

[11] Miszczak D., Cymerys J. (2014). A game of survival: Herpesvirus strategies of autophagy manipulation. Postepy Hig. Med. Dosw..

[12] Liang C.E.X., Jung J.U. (2008). Downregulation of autophagy by herpesvirus Bcl-2 homologs. Autophagy.

[13] Kvansakul M., Caria S., Hinds M.G. (2017). The Bcl-2 Family in Host-Virus Interactions. Viruses.

[14] Williams L.R., Taylor G.S. (2012). Autophagy and immunity-insights from human herpesviruses. Front. Immunol..

[15] Tognarelli E.I., Reyes A., Corrales N., Carreno L.J., Bueno S.M., Kalergis A.M., Gonzalez P.A. (2021). Modulation of Endosome Function, Vesicle Trafficking and Autophagy by Human Herpesviruses. Cells.

[16] Galluzzi L., Brenner C., Morselli E., Touat Z., Kroemer G. (2008). Viral control of mitochondrial apoptosis. PLoS Pathog..

[17] West A.P., Shadel G.S., Ghosh S. (2011). Mitochondria in innate immune responses. Nat. Rev. Immunol..

[18] Vazquez C., Horner S.M. (2015). MAVS Coordination of Antiviral Innate Immunity. J. Virol..

[19] Murphy M.P. (2009). How mitochondria produce reactive oxygen species. Biochem. J..

[20] Jounai N., Takeshita F., Kobiyama K., Sawano A., Miyawaki A., Xin K.Q., Ishii K.J., Kawai T., Akira S., Suzuki K. (2007). The Atg5 Atg12 conjugate associates with innate antiviral immune responses. Proc. Sci. Natl. Acad. Sci. USA.

[21] Tal M.C., Sasai M., Lee H.K., Yordy B., Shadel G.S., Iwasaki A. (2009). Absence of autophagy results in reactive oxygen species-dependent amplification of RLR signaling. Proc. Sci. Natl. Acad. Sci. USA.

[22] Choi Y.B., Harhaj E.W. (2014). Functional implications of mitochondrial reactive oxygen species generated by oncogenic viruses. Front. Biol..

[23] Wang R., Zhu Y., Ren C., Yang S., Tian S., Chen H., Jin M., Zhou H. (2020). Influenza A virus protein PB1-F2 impairs innate immunity by inducing mitophagy. Autophagy.

[24] Ding B., Zhang L., Li Z., Zhong Y., Tang Q., Qin Y., Chen M. (2017). The Matrix Protein of Human Parainfluenza Virus Type 3 Induces Mitophagy that Suppresses Interferon Responses. Cell Host Microbe..

[25] Wang K., Ma H., Liu H., Ye W., Li Z., Cheng L., Zhang L., Lei Y., Shen L., Zhang F. (2019). The Glycoprotein and Nucleocapsid Protein of Hantaviruses Manipulate Autophagy Flux to Restrain Host Innate Immune Responses. Cell Rep..

[26] Kim S.J., Syed G.H., Siddiqui A. (2013). Hepatitis C virus induces the mitochondrial translocation of Parkin and subsequent mitophagy. PLoS Pathog..

[27] Kim S.J., Syed G.H., Khan M., Chiu W.W., Sohail M.A., Gish R.G., Siddiqui A. (2014). Hepatitis C virus triggers mitochondrial fission and attenuates apoptosis to promote viral persistence. Proc. Sci. Natl. Acad. Sci. USA.

[28] Jassey A., Liu C.H., Changou C.A., Richardson C.D., Hsu H.Y., Lin L.T. (2019). Hepatitis C Virus Non-Structural Protein 5A (NS5A) Disrupts Mitochondrial Dynamics and Induces Mitophagy. Cells.

[29] Teodorof-Diedrich C., Spector S.A. (2018). Human Immunodeficiency Virus Type 1 gp120 and Tat Induce Mitochondrial Fragmentation and Incomplete Mitophagy in Human Neurons. J. Virol..

[30] Sin J., McIntyre L., Stotland A., Feuer R., Gottlieb R.A. (2017). Coxsackievirus B Escapes the Infected Cell in Ejected Mitophagosomes. J. Virol..

[31] Gou H., Zhao M., Xu H., Yuan J., He W., Zhu M., Ding H., Yi L., Chen J. (2017). CSFV induced mitochondrial fission and mitophagy to inhibit apoptosis. Oncotarget.

[32] Zhu L., Mou C., Yang X., Lin J., Yang Q. (2016). Mitophagy in TGEV infection counteracts oxidative stress and apoptosis. Oncotarget.

[33] Li S., Wang J., Zhou A., Khan F.A., Hu L., Zhang S. (2016). Porcine reproductive and respiratory syndrome virus triggers mitochondrial fission and mitophagy to attenuate apoptosis. Oncotarget.

[34] Meng G., Xia M., Wang D., Chen A., Wang Y., Wang H., Yu D., Wei J. (2014). Mitophagy promotes replication of oncolytic Newcastle disease virus by blocking intrinsic apoptosis in lung cancer cells. Oncotarget.

[35] Manzetti J., Weissbach F.H., Graf F.E., Unterstab G., Wernli M., Hopfer H., Drachenberg C.B., Rinaldo C.H., Hirsch H.H. (2020). BK Polyomavirus Evades Innate Immune Sensing by Disrupting the Mitochondrial Network and Promotes Mitophagy. iScience.

[36] Zhang Y., Sun R., Li X., Fang W. (2020). Porcine Circovirus 2 Induction of ROS Is Responsible for Mitophagy in PK-15 Cells via Activation of Drp1 Phosphorylation. Viruses.

[37] De Falco F., Urraro C., Cutarelli A., Roperto S. (2020). Bovine papillomavirus E5 oncoprotein upregulates parkin-dependent mitophagy in urothelial cells of cattle with spontaneous papillomavirus infection: A mechanistic study. Comp. Immunol. Microbiol. Infect. Dis..

[38] Roperto S., De Falco F., Perillo A., Catoi C., Roperto F. (2019). Mitophagy mediated by BNIP3 and BNIP3L/NIX in urothelial cells of the urinary bladder of cattle harbouring bovine papillomavirus infection. Vet. Microbiol..

[39] Roperto S., Russo V., De Falco F., Rosati A., Catoi C., Roperto F. (2019). FUNDC1-mediated mitophagy in bovine papillomavirus-infected urothelial cells. Vet. Microbiol..

[40] Kim S.J., Khan M., Quan J., Till A., Subramani S., Siddiqui A. (2013). Hepatitis B virus disrupts mitochondrial dynamics: Induces fission and mitophagy to attenuate apoptosis. PLoS Pathog..

[41] Vo M.T., Smith B.J., Nicholas J., Choi Y.B. (2019). Activation of NIX-mediated mitophagy by an interferon regulatory factor homologue of human herpesvirus. Nat. Commun..

[42] Palikaras K., Lionaki E., Tavernarakis N. (2018). Mechanisms of mitophagy in cellular homeostasis, physiology and pathology. Nat. Cell Biol..

[43] Lazarou M., Sliter D.A., Kane L.A., Sarraf S.A., Wang C., Burman J.L., Sideris D.P., Fogel A.I., Youle R.J. (2015). The ubiquitin kinase PINK1 recruits autophagy receptors to induce mitophagy. Nature.

[44] Heo J.M., Harper N.J., Paulo J.A., Li M., Xu Q., Coughlin M., Elledge S.J., Harper J.W. (2019). Integrated proteogenetic analysis reveals the landscape of a mitochondrial-autophagosome synapse during PARK2-dependent mitophagy. Sci. Adv..

[45] Novak I., Kirkin V., McEwan D.G., Zhang J., Wild P., Rozenknop A., Rogov V., Lohr F., Popovic D., Occhipinti A. (2010). Nix is a selective autophagy receptor for mitochondrial clearance. EMBO Rep..

[46] Band M., Joel A., Hernandez A., Avivi A. (2009). Hypoxia-induced BNIP3 expression and mitophagy: In vivo comparison of the rat and the hypoxia-tolerant mole rat, Spalax ehrenbergi. FASEB J..

[47] Liu L., Feng D., Chen G., Chen M., Zheng Q., Song P., Ma Q., Zhu C., Wang R., Qi W. (2012). Mitochondrial outer-membrane protein FUNDC1 mediates hypoxia-induced mitophagy in mammalian cells. Nat. Cell Biol..

[48] Wei Y., Chiang W.C., Sumpter R., Mishra P., Levine B. (2017). Prohibitin 2 Is an Inner Mitochondrial Membrane Mitophagy Receptor. Cell.

[49] Wertheim J.O., Smith M.D., Smith D.M., Scheffler K., Kosakovsky Pond S.L. (2014). Evolutionary origins of human herpes simplex viruses 1 and 2. Mol. Biol. Evol..

[50] Banerjee A., Kulkarni S., Mukherjee A. (2020). Herpes Simplex Virus: The Hostile Guest That Takes Over Your Home. Front. Microbiol..

[51] Cymerys J., Chodkowski M., Slonska A., Krzyzowska M., Banbura M.W. (2019). Disturbances of mitochondrial dynamics in cultured neurons infected with human herpesvirus type 1 and type 2. J. Neurovirol..

[52] Gershon A.A., Breuer J., Cohen J.I., Cohrs R.J., Gershon M.D., Gilden D., Grose C., Hambleton S., Kennedy P.G., Oxman M.N. (2015). Varicella zoster virus infection. Nat. Rev. Dis. Primers.

[53] Keller A.C., Badani H., McClatchey P.M., Baird N.L., Bowlin J.L., Bouchard R., Perng G.C., Reusch J.E., Kaufer B.B., Gilden D. (2016). Varicella zoster virus infection of human fetal lung cells alters mitochondrial morphology. J. Neurovirol..

[54] Young L.S., Yap L.F., Murray P.G. (2016). Epstein-Barr virus: More than 50 years old and still providing surprises. Nat. Rev. Cancer.

[55] Vilmen G., Glon D., Siracusano G., Lussignol M., Shao Z., Hernandez E., Perdiz D., Quignon F., Mouna L., Pous C. (2020). BHRF1, a BCL2 viral homolog, disturbs mitochondrial dynamics and stimulates mitophagy to dampen type I IFN induction. Autophagy.

[56] Song S., Jiang Z., Spezia-Lindner D.E., Liang T., Xu C., Wang H., Tian Y., Bai Y. (2020). BHRF1 Enhances EBV Mediated Nasopharyngeal Carcinoma Tumorigenesis through Modulating Mitophagy Associated with Mitochondrial Membrane Permeabilization Transition. Cells.

[57] Pal A.D., Basak N.P., Banerjee A.S., Banerjee S. (2014). Epstein-Barr virus latent membrane protein-2A alters mitochondrial dynamics promoting cellular migration mediated by Notch signaling pathway. Carcinogenesis.

[58] Griffiths P., Baraniak I., Reeves M. (2015). The pathogenesis of human cytomegalovirus. J. Pathol..

[59] Combs J.A., Norton E.B., Saifudeen Z.R., Bentrup K.H.Z., Katakam P.V., Morris C.A., Myers L., Kaur A., Sullivan D.E., Zwezdaryk K.J. (2020). Human Cytomegalovirus Alters Host Cell Mitochondrial Function during Acute Infection. J. Virol..

[60] Karniely S., Weekes M.P., Antrobus R., Rorbach J., van Haute L., Umrania Y., Smith D.L., Stanton R.J., Minczuk M., Lehner P.J. (2016). Human Cytomegalovirus Infection Upregulates the Mitochondrial Transcription and Translation Machineries. mBio.

[61] Bozidis P., Williamson C.D., Colberg-Poley A.M. (2007). Isolation of endoplasmic reticulum, mitochondria, and mitochondria-associated membrane fractions from transfected cells and from human cytomegalovirus-infected primary fibroblasts. Curr. Protoc. Cell Biol..

[62] Bhuvanendran S., Salka K., Rainey K., Sreetama S.C., Williams E., Leeker M., Prasad V., Boyd J., Patterson G.H., Jaiswal J.K. (2014). Superresolution imaging of human cytomegalovirus vMIA localization in sub-mitochondrial compartments. Viruses.

[63] Federspiel J.D., Cook K.C., Kennedy M.A., Venkatesh S.S., Otter C.J., Hofstadter W.A., Jean Beltran P.M., Cristea I.M. (2020). Mitochondria and Peroxisome Remodeling across Cytomegalovirus Infection Time Viewed through the Lens of Inter-ViSTA. Cell Rep..

[64] Zhang A., Hildreth R.L., Colberg-Poley A.M. (2013). Human cytomegalovirus inhibits apoptosis by proteasome-mediated degradation of Bax at endoplasmic reticulum-mitochondrion contacts. J. Virol..

[65] Hong C.T., Chau K.Y., Schapira A.H. (2016). The Cytomegalovirus protein pUL37x1 targets mitochondria to mediate neuroprotection. Sci. Rep..

[66] Carbone A., Gloghini A. (2008). KSHV/HHV8-associated lymphomas. Br. J. Haematol..

[67] Ganem D. (2006). KSHV infection and the pathogenesis of Kaposi’s sarcoma. Annu. Rev. Pathol..

[68] Cattelan A.M., Mattiolo A., Grassi A., Piano M.A., Sasset L., Trevenzoli M., Zanovello P., Calabro M.L. (2016). Predictors of immune reconstitution inflammatory syndrome associated with Kaposi’s sarcoma: A case report. Infect. Agent Cancer.

[69] Schulz T.F. (2006). The pleiotropic effects of Kaposi’s sarcoma herpesvirus. J. Pathol..

[70] Dai L., Trillo-Tinoco J., Bai L., Kang B., Xu Z., Wen X., Del Valle L., Qin Z. (2014). Systematic analysis of a xenograft mice model for KSHV+ primary effusion lymphoma (PEL). PLoS ONE.

[71] Hetz C., Zhang K., Kaufman R.J. (2020). Mechanisms, regulation and functions of the unfolded protein response. Nat. Rev. Mol. Cell Biol..

[72] Lamark T., Johansen T. (2012). Aggrephagy: Selective disposal of protein aggregates by macroautophagy. Int J. Cell Biol..

[73] Ichimura Y., Kirisako T., Takao T., Satomi Y., Shimonishi Y., Ishihara N., Mizushima N., Tanida I., Kominami E., Ohsumi M. (2000). A ubiquitin-like system mediates protein lipidation. Nature.

[74] Pankiv S., Clausen T.H., Lamark T., Brech A., Bruun J.A., Outzen H., Overvatn A., Bjorkoy G., Johansen T. (2007). p62/SQSTM1 binds directly to Atg8/LC3 to facilitate degradation of ubiquitinated protein aggregates by autophagy. J. Biol. Chem..

[75] Lu K., Psakhye I., Jentsch S. (2014). Autophagic clearance of polyQ proteins mediated by ubiquitin-Atg8 adaptors of the conserved CUET protein family. Cell.

[76] Kirkin V., Lamark T., Sou Y.S., Bjorkoy G., Nunn J.L., Bruun J.A., Shvets E., McEwan D.G., Clausen T.H., Wild P. (2009). A role for NBR1 in autophagosomal degradation of ubiquitinated substrates. Mol. Cell.

[77] Korac J., Schaeffer V., Kovacevic I., Clement A.M., Jungblut B., Behl C., Terzic J., Dikic I. (2013). Ubiquitin-independent function of optineurin in autophagic clearance of protein aggregates. J. Cell Sci..

[78] Filimonenko M., Isakson P., Finley K.D., Anderson M., Jeong H., Melia T.J., Bartlett B.J., Myers K.M., Birkeland H.C., Lamark T. (2010). The selective macroautophagic degradation of aggregated proteins requires the PI3P-binding protein Alfy. Mol. Cell.

[79] Clausen T.H., Lamark T., Isakson P., Finley K., Larsen K.B., Brech A., Overvatn A., Stenmark H., Bjorkoy G., Simonsen A. (2010). p62/SQSTM1 and ALFY interact to facilitate the formation of p62 bodies/ALIS and their degradation by autophagy. Autophagy.

[80] Lystad A.H., Ichimura Y., Takagi K., Yang Y., Pankiv S., Kanegae Y., Kageyama S., Suzuki M., Saito I., Mizushima T. (2014). Structural determinants in GABARAP required for the selective binding and recruitment of ALFY to LC3B-positive structures. EMBO Rep..

[81] Simonsen A., Birkeland H.C., Gillooly D.J., Mizushima N., Kuma A., Yoshimori T., Slagsvold T., Brech A., Stenmark H. (2004). Alfy, a novel FYVE-domain-containing protein associated with protein granules and autophagic membranes. J. Cell Sci..

[82] Lu K., den Brave F., Jentsch S. (2017). Receptor oligomerization guides pathway choice between proteasomal and autophagic degradation. Nat. Cell Biol..

[83] Wurzer B., Zaffagnini G., Fracchiolla D., Turco E., Abert C., Romanov J., Martens S. (2015). Oligomerization of p62 allows for selection of ubiquitinated cargo and isolation membrane during selective autophagy. eLife.

[84] Korolchuk V.I., Menzies F.M., Rubinsztein D.C. (2010). Mechanisms of cross-talk between the ubiquitin-proteasome and autophagy-lysosome systems. FEBS Lett..

[85] Brune W., Ménard C., Heesemann J., Koszinowski U.H. (2001). A ribonucleotide reductase homolog of cytomegalovirus and endothelial cell tropism. Science.

[86] Muscolino E., Schmitz R., Loroch S., Caragliano E., Schneider C., Rizzato M., Kim Y.H., Krause E., Juranic Lisnic V., Sickmann A. (2020). Herpesviruses induce aggregation and selective autophagy of host signalling proteins NEMO and RIPK1 as an immune-evasion mechanism. Nat. Microbiol..

[87] Aghi M., Visted T., Depinho R.A., Chiocca E.A. (2008). Oncolytic herpes virus with defective ICP6 specifically replicates in quiescent cells with homozygous genetic mutations in p16. Oncogene.

[88] Park Y.E., Hayashi Y.K., Bonne G., Arimura T., Noguchi S., Nonaka I., Nishino I. (2009). Autophagic degradation of nuclear components in mammalian cells. Autophagy.

[89] Shoji J.Y., Kikuma T., Arioka M., Kitamoto K. (2010). Macroautophagy-mediated degradation of whole nuclei in the filamentous fungus Aspergillus oryzae. PLoS ONE.

[90] Changou C.A., Chen Y.R., Xing L., Yen Y., Chuang F.Y., Cheng R.H., Bold R.J., Ann D.K., Kung H.J. (2014). Arginine starvation-associated atypical cellular death involves mitochondrial dysfunction, nuclear DNA leakage, and chromatin autophagy. Proc. Sci. Natl. Acad. Sci. USA.

[91] Dou Z., Xu C., Donahue G., Shimi T., Pan J.A., Zhu J., Ivanov A., Capell B.C., Drake A.M., Shah P.P. (2015). Autophagy mediates degradation of nuclear lamina. Nature.

[92] Paludan C., Schmid D., Landthaler M., Vockerodt M., Kube D., Tuschl T., Munz C. (2005). Endogenous MHC class II processing of a viral nuclear antigen after autophagy. Science.

[93] Gonnella R., Dimarco M., Farina G.A., Santarelli R., Valia S., Faggioni A., Angeloni A., Cirone M., Farina A. (2020). BFRF1 protein is involved in EBV-mediated autophagy manipulation. Microbes Infect..

[94] Angeloni A., Farina A., Gentile G., Capobianchi A., Martino P., Visco V., Muraro R., Frati L., Faggioni A. (2001). Epstein-Barr virus and breast cancer: Search for antibodies to the novel BFRF1 protein in sera of breast cancer patients. J. Natl. Cancer Inst..

[95] Farina A., Santarelli R., Gonnella R., Bei R., Muraro R., Cardinali G., Uccini S., Ragona G., Frati L., Faggioni A. (2000). The BFRF1 gene of Epstein-Barr virus encodes a novel protein. J. Virol..

[96] Gonnella R., Farina A., Santarelli R., Raffa S., Feederle R., Bei R., Granato M., Modesti A., Frati L., Delecluse H.J. (2005). Characterization and intracellular localization of the Epstein-Barr virus protein BFLF2: Interactions with BFRF1 and with the nuclear lamina. J. Virol..

[97] Liu G.T., Kung H.N., Chen C.K., Huang C., Wang Y.L., Yu C.P., Lee C.P. (2018). Improving nuclear envelope dynamics by EBV BFRF1 facilitates intranuclear component clearance through autophagy. FASEB J..

[98] Farina A., Feederle R., Raffa S., Gonnella R., Santarelli R., Frati L., Angeloni A., Torrisi M.R., Faggioni A., Delecluse H.J. (2005). BFRF1 of Epstein-Barr virus is essential for efficient primary viral envelopment and egress. J. Virol..

[99] Turan A., Grosche L., Krawczyk A., Muhl-Zurbes P., Drassner C., Duthorn A., Kummer M., Hasenberg M., Voortmann S., Jastrow H. (2019). Autophagic degradation of lamins facilitates the nuclear egress of herpes simplex virus type 1. J. Cell Biol..

[100] Kaur J., Debnath J. (2015). Autophagy at the crossroads of catabolism and anabolism. Nat. Rev. Mol. Cell Biol..

[101] Pantopoulos K., Porwal S.K., Tartakoff A., Devireddy L. (2012). Mechanisms of mammalian iron homeostasis. Biochemistry.

[102] Mancias J.D., Pontano Vaites L., Nissim S., Biancur D.E., Kim A.J., Wang X., Liu Y., Goessling W., Kimmelman A.C., Harper J.W. (2015). Ferritinophagy via NCOA4 is required for erythropoiesis and is regulated by iron dependent HERC2-mediated proteolysis. eLife.

[103] Goodwin J.M., Dowdle W.E., DeJesus R., Wang Z., Bergman P., Kobylarz M., Lindeman A., Xavier R.J., McAllister G., Nyfeler B. (2017). Autophagy-Independent Lysosomal Targeting Regulated by ULK1/2-FIP200 and ATG9. Cell Rep..

[104] Sun Y., Bao Q., Xuan B., Xu W., Pan D., Li Q., Qian Z. (2018). Human Cytomegalovirus Protein pUL38 Prevents Premature Cell Death by Binding to Ubiquitin-Specific Protease 24 and Regulating Iron Metabolism. J. Virol..

[105] Gomes L.C., Dikic I. (2014). Autophagy in antimicrobial immunity. Mol. Cell.

[106] Thurston T.L., Ryzhakov G., Bloor S., von Muhlinen N., Randow F. (2009). The TBK1 adaptor and autophagy receptor NDP52 restricts the proliferation of ubiquitin-coated bacteria. Nat. Immunol..

[107] Zheng Y.T., Shahnazari S., Brech A., Lamark T., Johansen T., Brumell J.H. (2009). The adaptor protein p62/SQSTM1 targets invading bacteria to the autophagy pathway. J. Immunol..

[108] Thurston T.L., Wandel M.P., von Muhlinen N., Foeglein A., Randow F. (2012). Galectin 8 targets damaged vesicles for autophagy to defend cells against bacterial invasion. Nature.

[109] Sumpter R., Sirasanagandla S., Fernández Á., Wei Y., Dong X., Franco L., Zou Z., Marchal C., Lee M.Y., Clapp D.W. (2016). Fanconi Anemia Proteins Function in Mitophagy and Immunity. Cell.

[110] Orvedahl A., Sumpter R., Xiao G., Ng A., Zou Z., Tang Y., Narimatsu M., Gilpin C., Sun Q., Roth M. (2011). Image-based genome-wide siRNA screen identifies selective autophagy factors. Nature.

[111] Dong X., Yang Y., Zou Z., Zhao Y., Ci B., Zhong L., Bhave M., Wang L., Kuo Y.C., Zang X. (2021). Sorting nexin 5 mediates virus-induced autophagy and immunity. Nature.

[112] Hase K., Fujiwara Y., Kikuchi H., Aizawa S., Hakuno F., Takahashi S., Wada K., Kabuta T. (2015). RNautophagy/DNautophagy possesses selectivity for RNA/DNA substrates. Nucleic Acids Res..

[113] Hase K., Contu V.R., Kabuta C., Sakai R., Takahashi M., Kataoka N., Hakuno F., Takahashi S.I., Fujiwara Y., Wada K. (2020). Cytosolic domain of SIDT2 carries an arginine-rich motif that binds to RNA/DNA and is important for the direct transport of nucleic acids into lysosomes. Autophagy.

[114] Fujiwara Y., Hase K., Wada K., Kabuta T. (2015). An RNautophagy/DNautophagy receptor, LAMP2C, possesses an arginine-rich motif that mediates RNA/DNA-binding. Biochem. Biophys. Res. Commun..

[115] Rasmussen S.B., Horan K.A., Holm C.K., Stranks A.J., Mettenleiter T.C., Simon A.K., Jensen S.B., Rixon F.J., He B., Paludan S.R. (2011). Activation of autophagy by alpha-herpesviruses in myeloid cells is mediated by cytoplasmic viral DNA through a mechanism dependent on stimulator of IFN genes. J. Immunol..

[116] Motwani M., Pesiridis S., Fitzgerald K.A. (2019). DNA sensing by the cGAS-STING pathway in health and disease. Nat. Rev. Genet..

[117] Gui X., Yang H., Li T., Tan X., Shi P., Li M., Du F., Chen Z.J. (2019). Autophagy induction via STING trafficking is a primordial function of the cGAS pathway. Nature.

[118] Liu D., Wu H., Wang C., Li Y., Tian H., Siraj S., Sehgal S.A., Wang X., Wang J., Shang Y. (2019). STING directly activates autophagy to tune the innate immune response. Cell Death Differ..

[119] Ahmad L., Mashbat B., Leung C., Brookes C., Hamad S., Krokowski S., Shenoy A.R., Lorenzo L., Levin M., O’Hare P. (2019). Human TANK-binding kinase 1 is required for early autophagy induction upon herpes simplex virus 1 infection. J. Allergy Clin. Immunol. Immunol..

[120] Sparrer K.M.J., Gableske S., Zurenski M.A., Parker Z.M., Full F., Baumgart G.J., Kato J., Pacheco-Rodriguez G., Liang C., Pornillos O. (2017). TRIM23 mediates virus-induced autophagy via activation of TBK1. Nat. Microbiol..

[121] Pilli M., Arko-Mensah J., Ponpuak M., Roberts E., Master S., Mandell M.A., Dupont N., Ornatowski W., Jiang S., Bradfute S.B. (2012). TBK-1 promotes autophagy-mediated antimicrobial defense by controlling autophagosome maturation. Immunity.

[122] Talloczy Z., Jiang W., Virgin H.W.t., Leib D.A., Scheuner D., Kaufman R.J., Eskelinen E.L., Levine B. (2002). Regulation of starvation- and virus-induced autophagy by the eIF2alpha kinase signaling pathway. Proc. Sci. Natl. Acad. Sci. USA.

[123] Orvedahl A., Alexander D., Talloczy Z., Sun Q., Wei Y., Zhang W., Burns D., Leib D.A., Levine B. (2007). HSV-1 ICP34.5 confers neurovirulence by targeting the Beclin 1 autophagy protein. Cell Host Microbe.

[124] Lussignol M., Queval C., Bernet-Camard M.F., Cotte-Laffitte J., Beau I., Codogno P., Esclatine A. (2013). The herpes simplex virus 1 Us11 protein inhibits autophagy through its interaction with the protein kinase PKR. J. Virol..

[125] Waisner H., Kalamvoki M. (2019). The ICP0 Protein of Herpes Simplex Virus 1 (HSV-1) Downregulates Major Autophagy Adaptor Proteins Sequestosome 1 and Optineurin during the Early Stages of HSV-1 Infection. J. Virol..

[126] Liu X., Matrenec R., Gack M.U., He B. (2019). Disassembly of the TRIM23-TBK1 Complex by the Us11 Protein of Herpes Simplex Virus 1 Impairs Autophagy. J. Virol..

[127] Chaumorcel M., Lussignol M., Mouna L., Cavignac Y., Fahie K., Cotte-Laffitte J., Geballe A., Brune W., Beau I., Codogno P. (2012). The human cytomegalovirus protein TRS1 inhibits autophagy via its interaction with Beclin 1. J. Virol..

[128] Lee J.S., Li Q., Lee J.Y., Lee S.H., Jeong J.H., Lee H.R., Chang H., Zhou F.C., Gao S.J., Liang C. (2009). FLIP-mediated autophagy regulation in cell death control. Nat. Cell Biol..

[129] Pattingre S., Tassa A., Qu X., Garuti R., Liang X.H., Mizushima N., Packer M., Schneider M.D., Levine B. (2005). Bcl-2 antiapoptotic proteins inhibit Beclin 1-dependent autophagy. Cell.

